# Is there something new regarding triceps brachii muscle insertion?^1^

**DOI:** 10.1590/s0102-865020200100000007

**Published:** 2020-11-23

**Authors:** Flávia Emi Akamatsu, José Renato Negrão, Marcelo Bordalo Rodrigues, Ana Maria Itezerote, Samir Omar Saleh, Flávio Hojaij, Mauro Andrade, Alfredo Luiz Jacomo

**Affiliations:** I PhD, Department of Surgery, Laboratory of Medical Research 02 - Division of Human Structural Topography, Faculdade de Medicina, Universidade de São Paulo (FMUSP), Brazil. Intellectual and scientific content of the study, acquisition of data, technical procedures, manuscript writing, final approval.; II MD, Radiology Institute (INRAD), Clinics Hospital, FMUSP, Sao Paulo-SP, Brazil. Design, intellectual and scientific content of the study; acquisition of data; technical procedures; manuscript writing; final approval.; III MD, PhD, INRAD, Clinics Hospital, FMUSP, Sao Paulo-SP, Brazil. Technical procedures, interpretation of data, final approval.; IV PhD, Department of Surgery, Laboratory of Medical Research 02 - Division of Human Structural Topography, FMUSP, Sao Paulo-SP, Brazil. Acquisition of data, technical procedures, final approval.; V MD, PhD, Department of Surgery, Laboratory of Medical Research 02 - Division of Human Structural Topography, FMUSP, Sao Paulo-SP, Brazil. Technical procedure, interpretation of data, final approval.; VI PhD, Department of Surgery, Laboratory of Medical Research 02 - Division of Human Structural Topography, FMUSP, Sao Paulo-SP, Brazil. Interpretation of data, manuscript writing, critical revision, final approval.; VII PhD, Department of Surgery, Laboratory of Medical Research 02 - Division of Human Structural Topography, FMUSP, Sao Paulo-SP Brazil. Conception, intellectual and scientific content of the study; critical revision; final approval.

**Keywords:** Tendons, Muscles, Magnetic Resonance Imaging, Anatomy

## Abstract

**Purpose:**

Previous studies have questioned whether the triceps brachii muscle tendon (TBMT) has a double or single insertion on the ulna. Aiming to provide an answer, we describe the anatomy of the TBMT and review a magnetic resonance imaging (MRI) series of the elbow.

**Methods:**

Forty-one elbows were dissected to assess the details of the triceps brachii insertion. Elbow plastination slices were analyzed to determine whether there was a space on the TBMT. Magnetic resonance imaging from the records of the authors were also obtained to demonstrate the appearance of the pre-tricipital space on MRI.

**Results:**

A virtual space on the medial aspect near the TBTM insertion site in the olecranon was consistently found on anatomic dissections. It was a distal pre-tricipital space. Magnetic resonance imaging demonstrated the appearance of the pre-tricipital space on MRI, and its extension was measured longitudinally either in elbow flexion or extension. There was no statistically significant difference between the measurements of this space in the right and left elbows or between flexion and extension (p > 0.05). The coefficient of variation was <10% for all measurements.

**Conclusion:**

Knowledge of this structure may be essential to avoid incorrect diagnosis and unnecessary therapeutic interventions.

## Introduction

The anatomical characterization of the insertion of the triceps brachii muscle tendon (TBMT) on the olecranon is very important for clinical and surgical approaches in terms of elbow trauma and tendon repair, and it has been the subject of research for decades^1^ ^-^ ^5^. Injuries to the distal triceps muscle are uncommon, and their diagnosis is challenging and strongly dependent on imaging methods^3^ ^,^ ^5^ ^,^ ^6^. The prevalence of triceps tendon injuries has been found to be 3.8%^7^. Albeit uncommon, this condition can cause significant symptoms. Complete loss of extension is seen in 20% of cases regarding tendon rupture, although intact lateral expansion of the tendon may allow some active extension, possibly leading to diagnostic error^4^.

Previous studies have questioned whether the TBMT has a double or single insertion on the ulna, referring to MRI that suggests a bipartite TBMT insertion. Anatomical and histological evaluation has proven elusive, with controversial results^8^ ^-^ ^10^. The bipartite aspect of the tendon that was identified on MRI was not seen by histologic study^8^.

Among imaging techniques, MRI provides superior resolution of soft-tissue contrast, no exposure to ionizing radiation, good reproducibility, noninvasiveness, and multiplanar sections, thus making it particularly useful in assessing TBMT insertion anatomy^11^ ^-^ ^13^.

We aimed to describe the anatomy of the TBMT and assess its correlation with the bipartite images observed on MRI, considering the importance of proper anatomic characterization of this structure for the clinical and surgical management of elbow trauma. Detailed knowledge of TBMT anatomy permits adequate evaluation and interpretation by radiologists to provide important information to orthopedic surgeons in cases of potential tendon lesions.

## Methods

This study was approved by the University Research Ethics Committee under protocol 216/14.

### Anatomical dissection

**Figure 1:**
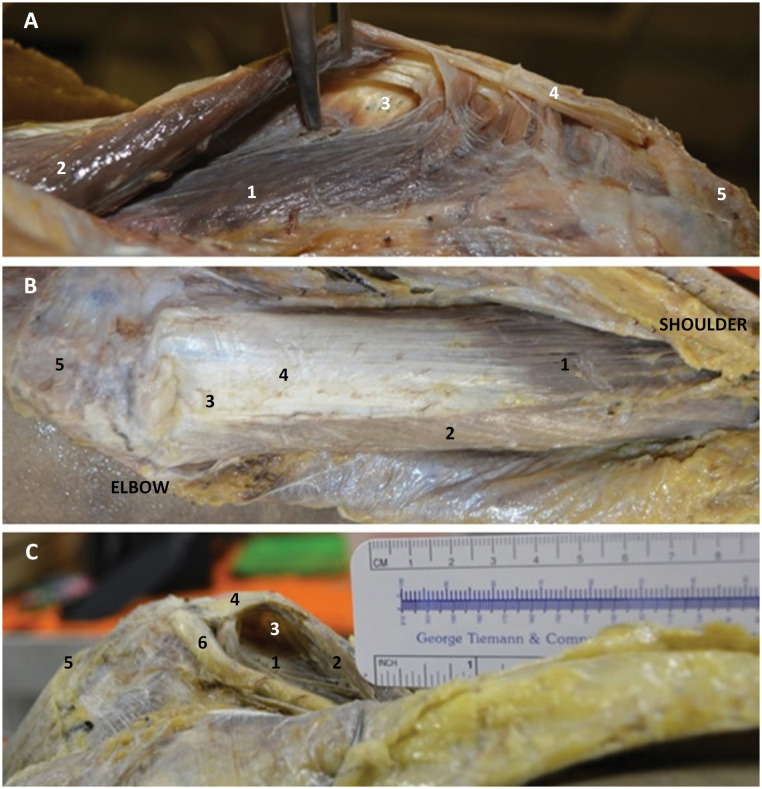
Left upper extremity. **A**) Lateral view of the TBMT insertion. 1, medial head of the triceps; 2, lateral head of the triceps; 3, open plane; 4, TBMT; 5, olecranon. **B**) Posterior view. 1, lateral head of the triceps; 2, long head of the triceps; 3, strip; 4, TBMT; 5, olecranon. **C**) Medial view of the TBMT insertion. 1, medial head of the triceps; 2, long head of the triceps; 3, open plane; 4, TBMT; 5, olecranon; 6, ulnar nerve.

Our study aimed to observe the structural details of the insertion of the triceps brachii muscle onto the olecranon by direct anatomical dissection of adult cadavers. Forty-one elbows from human adult cadavers (33 from males, 8 from females) were dissected to expose the TBMT. The cadavers were fixed using a 4% phenolic acid and 0.5% formaldehyde solution and were obtained from a body donation program undertaken by the Discipline of Human Structural Topography of the Department of Surgery of the University. Specimens with no sign of previous surgery or any other severe abnormality in the regions of interest were included. The specimens were placed in a ventral decubitus position on the dissection table, with the arms positioned at approximately 30 degrees of elbow flexion. A medial incision was made starting from 10 centimeters above the olecranon and continuing 5 centimeters down this structure. Next, flaps of skin and subcutaneous tissue were retracted to expose the triceps brachii muscle, which was kept in its anatomical position (Fig. 1). The tendons of the long, lateral, and medial heads were dissected to observe their insertions. A curved incision with the concavity facing the cranial side was made to expose the olecranon, and the plane between the common tendon and the medial humeral condyle was accessed.

Measurements were made with digital calipers. Photographic documentation was captured with a Nikon D5200™ digital single-lens reflex camera (Nikon, Japan).

### Plastination slices

Plastination slices used in laboratory for practical classes to our graduate students were reviewed to compare with gross anatomy findings and MRI to ascertain whether plastination methods could modify them.

Sagittal and coronal plastination slices of 6 elbows obtained from the Discipline of Human Structural Topography were used for comparative analysis.

### MRI

We tried to correlate anatomical findings with MRI normal elbows obtained from the general archive of the Department of Radiology of our hospital.

Magnetic resonance imaging from the records of the authors were also obtained to evaluate the region of interest.

A total of 21 elbow MRI scans were randomly selected from patients (13 males, 8 females; age range from 11-77) who underwent imaging to evaluate clinical symptoms suggestive of epicondylitis and extensor tendinitis and who had no history of elbow surgery or trauma.

All MRI studies were performed with a 1.5 T unit (HDX, GE^™^). A knee coil was employed. The elbow was placed in extension in the center of the gantry, and sagittal, coronal and axial T1- and T2-weighted spin-echo MRI (3.0 mm section thickness, 0.5 intersection space, 13×13 cm field of view, 384×224 and 320×192 matrix size) were obtained.

### Statistical analysis

Student's t-test was used in Anatomical dissection to compare measurements obtained from males and females (α = 0.05). Pearson correlation coefficients were used for length and other discrete variables (sex, age and side). Data are presented as the means and 95% confidence intervals (95% CIs). Statistical significance was accepted at p < 0.05. Scans of MRI were assessed by three independent observers, who were all experienced radiologists from the Orthopedics Institute of the University, School of Medicine.

Variables were described using the patients’ absolute and relative frequencies and age as well as through standard measures of central tendency and dispersion (mean, median, standard deviation, and range) (Table 1). Fleiss Kappa coefficients, with the respective 95% CIs^14^ were calculated to assess observers´ agreement in MRI interpretation. Data were compiled in Microsoft Excel 2010 and analyzed in IBM SPSS Statistics for Windows, Version 22.0.

**Table 1:** Description of the patients who underwent magnetic resonance imaging (MRI) of the elbow.

Variable	(N=21)
Age (years)	
Mean (SD)	37.7 (17.4)
Median (min; max)	34 (11; 77)
**Sex**	**N (%)**
Female	8 (38.1)
Male	13 (61.9)
**Side**	**N (%)**
Right	13 (61.9)
Left	8 (38.1)

## Results

### Anatomic dissection

**Figure 2:**
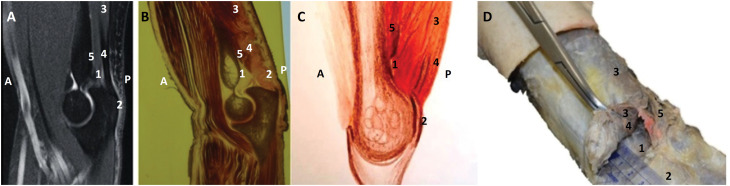
Left upper extremity: **A** and **B**) Medial sagittal slices. **A**, MRI; **B**, Plastination specimen. A, anterior; P, posterior; 1, distal pre-tricipital space; 2, common triceps brachii tendon; 3, long head of the triceps; 4, tendon of the long head of the triceps; 5, medial head of the triceps. **C**) Schematic diagram. A, anterior; P, posterior; 1, distal pre-tricipital space; 2, common triceps brachii tendon; 3, long head of the triceps; 4, long head tendon; 5, medial head of the triceps. **D**) Dissection, posterior view. 1, distal pre-tricipital space; 2, olecranon; 3, long head of the triceps; 4, common triceps brachii tendon; 5, ulnar nerve.

**Figure 3:**
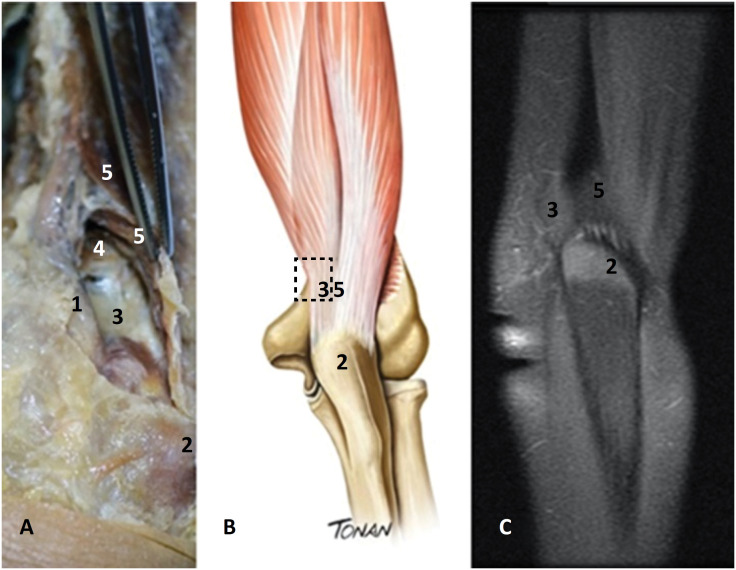
Right upper extremity. **A**) Photo of a cadaveric specimen. Posterior view, near the olecranon insertion: 1, ulnar nerve; 2, olecranon; 3, distal pre-tricipital space; 4, muscle fibers of the medial head of the triceps; 5, tendon and muscle fibers of the long head of the triceps (strip). **B**) Schematic diagram (Source-Rodrigo Tonan) 2, olecranon; 3, distal pre-tricipital space; 5, long head of the triceps tendon. **C**) MRI, coronal slice. 2, olecranon; 3, distal pre-tricipital space; 5, tendon of the long head of the triceps.

A virtual space on the medial aspect near the TBTM insertion site on the olecranon was consistently found on anatomic dissections (Figs. 2 and 3). It was a distal pre-tricipital space. The fibers of the medial head inserted proximal to the medial condyle of the humerus on the medial aspect, while laterally, its fibers reached the lateral condyle (Fig. 1 A,C). This distal pre-tricipital space was located over the medial condyle, immediately distal to the medial head of the muscle (Figs. 2 and 3). The tendon of the long head was found to cover this space as a thin and translucent strip (Figs. 1B and 3B). This distal pre-tricipital space was occupied by connective tissue, suggesting a bursa or sac (Figs. 2 and 3). It was bounded superiorly by the medial head of the muscle; laterally by the common deep tendon and fibers of the medial head, inserting onto the lateral condyle; and medially by the tendon of the medial head and the ulnar nerve and inferiorly by the joint capsule. The floor of this space was represented by the medial condyle, while its roof was composed of the fibers and tendon of the long head of the muscle as well as the common tendon.

This area was located approximately 4.3±0.35 cm (males) and 4.4±0.45 cm (females) from the olecranon (Fig. 1B) and was 1.5 cm long. It did not have a defined shape. We measured the longitudinal dimension from the olecranon to the superior boundary and from the superior to the inferior boundaries of this area and found a coefficient of variation of less than 10% for all measurements. The region of the tendon of the long head (before joining the tendons of the lateral and medial heads) was visible as a superficial, translucent blade of the long head of the TBMT (Fig. 1C). This thin, translucent, strip-like area measured 1.0 cm in diameter and 3.0 cm in length and was located 5.0 cm from the olecranon.

### Plastination slices

In all elbow plastination slices (sagittal and coronal), the space described above was found, as it was found in the anatomical dissections.

### MRI

Magnetic resonance imaging also demonstrated the appearance of the pre-tricipital space near the olecranon.

Of all three observers (experienced musculoskeletal radiologists), only one did not agree that a space as described above was present on the MRI (Table 2).

**Table 2:** Fleiss Kappa coefficients of interobserver agreement as to the presence of the triceps brachii space on MRI of the elbow. The Fleiss kappa score was close to zero (0.138) and the confidence interval crossed zero (-0.109 to 0.385).

MRI	Observer1	Observer2	Observer3	Fleiss Kappa
				IC (95%)
Negative	10 (47.6)	6 (28.6)	9 (42.9)	0.138
Positive	11 (52.4)	15 (71.4)	12 (57.1)	(-0.109; 0.385)
**Total**	**21 (100)**	**21 (100)**	**21 (100)**	

Data expressed as n (%)

## Discussion

Belentani *et al*.^8^ reported the use of sequential MRI scans and histological analyses to determine the insertion of the TBMT in cadavers. In their series, insertion images demonstrated a split pattern, not confirmed by histological evaluation, which found it to be a single insertion^8^. In our retrospective analysis of MRI scans, we identified a hyperintense area on T1-weighted spin-echo images on the lateral aspect of the medial supracondylar crest.

A distal pre-tricipital space is found on anatomical dissections, which has probably been previously interpreted as a division, giving the TBMT insertion a bipartite appearance as described previously in the literature^3^ ^,^ ^5^ ^-^ ^6^. In some MRIs from our records, observers suggested the existence of a pre-tricipital space, even though agreement between observers was poor. Further studies are needed to confirm these findings. Kappa, for Fleiss were close to zero because there was disagreement between observers and not only because the sample was small. There was a disagreement between observers, probably due to the fact that the pre-tricipital space has not, until now, been described anatomically. The study was perhaps a first analysis of this finding. The study would be the first study to assess the pre-tricipital space using MRI.

The anatomy of the distal portion of the TBMT has received little attention in the orthopedic literature^9^. The triceps brachii muscle is the main extensor of the forearm and the main antagonist of the brachialis and biceps brachii muscles. The origins of the three heads are different; however, their insertions have been studied, since the medial head is slightly more separated from the long and lateral heads, representing a more bipartite aspect^8^. According to the anatomy book Gray's Anatomy^15^, the triceps brachii muscle occupies most of the extensor compartment of the arm and, as its name implies, consists of three heads (long, lateral, and medial). The long head originates as a flattened tendon from the infraglenoid tubercle of the scapula, fusing with the capsule of the shoulder joint. Its muscular fibers descend medially to the lateral head and superficial to the medial head, joining to form a common tendon. The lateral head originates as a flat tendon from a narrow, linear and oblique crest on the posterior surface of the humeral shaft and lateral intermuscular septum. The humeral origin runs obliquely above and lateral to the groove of the radial nerve and proximal to the deltoid tuberosity on the medial aspect of the surgical neck. These fibers also converge as the common tendon. The medial head, which lies deep to the lateral and long heads, has a particularly long attachment to the posterior aspect of the humeral shaft. It extends below the radial groove from the insertion of the teres major muscle to within 2.5 cm of the trochlea, the medial margin of the humerus, the medial intermuscular septum, and the lower part of the lateral intermuscular septum. Some muscle fibers insert onto the olecranon, whereas the remaining fibers form part of the common tendon. The TBMT begins approximately at the midpoint of the muscle and has two layers, superficial and deep. After receiving the muscle fibers, the two layers attach mostly onto the tip of the olecranon. On the lateral side, a band of fibers continues down onto the anconeus muscle to merge with the fascia of the forearm^15^.

In this study, we found a distal pre-tricipital space at the distal portion of the TBMT in all dissected specimens. To the best of our knowledge, no such space has ever been described in the literature. This distal pre-tricipital space is located on the medial aspect of the TBMT insertion before it reaches the olecranon, lateral to the supracondylar crest. Its appearance resembles that of a bursa or sac, which suggests that fluid can build up within the space. This space was clearly present in our plastination specimens, even though it has not been described before. We believe that this feature corresponds to the bipartite appearance of the TBMT as it inserts onto the olecranon on MRI scans. Knowledge of the deep and superficial anatomy of the triceps tendon is important in the MRI assessment of tendon injuries since the medial head of the TBMT can rupture in isolation, causing a reduction in the strength of the extensor mechanism though the incomplete loss of function; this pattern of injury generally occurs in weightlifting athletes^10^. The distal pre-tricipital space we describe herein may play an important role in TBMT lesions because it can accumulate fluid, and it should be considered in patients presenting with elbow complaints. During the course of the triceps tendon dissection, we observed that the tendon of the long head appeared as a very thin, translucent structure before forming the proper TBMT. This translucent area lies superficial to the head of the medial triceps muscle, not to its tendon, and also overlies the distal pre-tricipital space. It has the shape of a strip and is observable before the tendon inserts onto the olecranon, not in the crest of the ulna, as reported by Celli^3^. In our sample, it was present with a 100% incidence near the attachment of the fibers of the long head of the triceps, forming the roof of the distal pre-tricipital space we describe. This structure has also been previously undescribed in the literature, probably due to its proximity to other well-known structures such as the medial head of the triceps brachii muscle (which lies inferior to this area), the tendon of the lateral head of the triceps (lateral to this area) and the tendon of the long head of the triceps. During layered dissection of this region, lifting the muscle away modifies the arrangement of the three heads, making the structures we describe difficult to visualize. Thus, we attempted to dissect the structures only by separating the tissue planes and found the clearly demarcated space we now describe. The thinner, translucent part of the long head of the tendon certainly makes it more vulnerable to lesions. A deep tendon separated from a more superficial tendon was reported by Madsen *et al*.^10^.

We suggest that the bipartite aspect of the TBMT on MRI previously described in the literature^8^ may represent the plane formed under this translucent area of the long head of the triceps tendon. The well-defined interval between the lateral triceps expansion and medial triceps tendon just proximal to the olecranon along the crest of the ulna, known as the “triceps decussation”^3^, could be the plane found in our scans. One report suggests that the TBMT inserts as a bilaminar tendon over a wide area onto the tip of the olecranon^5^. Nevertheless, we found only a single insertion of the TBMT on the olecranon.

The distal pre-tricipital space we describe, when filled with fluid in the presence of TBMT injury or inflammation, can likely take on an imaging aspect that leads to misinterpretation of its actual anatomic nature. Shuttlewood *et al*.^5^ stressed that full-thickness tears of the TBMT are usually easily diagnosed on clinical examination, but partial-thickness tears can be missed and require imaging with either MRI or ultrasound. We suggest that this thinner, translucent area of the tendon may be more vulnerable to injury and, when implicated in partial-thickness lesions, may make them more difficult to diagnose. Heikenfeld *et al*.^16^ reported that partial tears of the superficial layer of the distal triceps tendon are probably more common than expected and not always associated with a clear history of trauma. Partial tears can be located on the superficial tendon head only (combined lateral or long)^12^ ^,^ ^16^ ^-^ ^17^ Magnetic resonance imaging can demonstrate the shape and site of the rupture as well as determine the most appropriate treatment method^18^. Imaging is crucial for correct diagnosis for clinical diagnosis of this type of injury is difficult and often inaccurate, especially in acute settings^4^ ^,^ ^7^ ^,^ ^19^. This distal pre-tricipital space could be considered an artifact or anatomical lesion, but the anatomical finding have shown that it is a space between three heads in the most distal region close to insertion into the olecranon. This finding can help to elucidate the previous research conflict about the image that appear in MRI since other articles not exactly described this region. Correct recognition of the distal pre-tricipital space may avoid misdiagnosis of a suspected elbow lesion.

Despite the small sample size in this study, anatomical findings are relevant and were observed in all our cadaveric speciments and plastination slices. This previously undescribed space may account for some misdiagnosis in imaging studies of the elbow and correlation with MRI should contribute to the correct interpretation of the images.

## Conclusion

Knowledge of this structure may be essential to avoid incorrect diagnosis and unnecessary therapeutic interventions.
